# Comparison of Two Molecular Assays for Detection and Characterization of *Aspergillus fumigatus* Triazole Resistance and *Cyp51A* Mutations in Clinical Isolates and Primary Clinical Samples of Immunocompromised Patients

**DOI:** 10.3389/fmicb.2018.00555

**Published:** 2018-03-27

**Authors:** Patricia Postina, Julian Skladny, Tobias Boch, Oliver A. Cornely, Axel Hamprecht, Peter-Michael Rath, Jörg Steinmann, Oliver Bader, Thomas Miethke, Anne Dietz, Natalia Merker, Wolf-Karsten Hofmann, Dieter Buchheidt, Birgit Spiess

**Affiliations:** ^1^Department of Hematology and Oncology, University Hospital Mannheim, Heidelberg University, Mannheim, Germany; ^2^Department I of Internal Medicine, University Hospital of Cologne, Cologne, Germany; ^3^Clinical Trials Centre Cologne, ZKS Köln and Cologne Excellence Cluster on Cellular Stress Response in Aging-Associated Diseases, University of Cologne, Cologne, Germany; ^4^Institute of Medical Microbiology, Immunology and Hygiene, University Hospital of Cologne, Cologne, Germany; ^5^Institute of Medical Microbiology, University Hospital Essen, Essen, Germany; ^6^Institute of Clinical Hygiene, Medical Microbiology and Clinical Infectiology, Paracelsus Medical University, Nuremberg, Germany; ^7^Institute for Medical Microbiology, University Medical Center Göttingen, Göttingen, Germany; ^8^Institute of Medical Microbiology and Hygiene, University Hospital Mannheim, Heidelberg University, Mannheim, Germany

**Keywords:** invasive aspergillosis, triazole resistance, PCR, clinical samples, melting curve analysis

## Abstract

In hematological patients, the incidence of invasive aspergillosis (IA) caused by azole resistant *Aspergillus fumigatus* (ARAf) is rising. As the diagnosis of IA is rarely based on positive culture in this group of patients, molecular detection of resistance mutations directly from clinical samples is crucial. In addition to the in-house azole resistance ARAf polymerase chain reaction (PCR) assays detecting the frequent mutation combinations TR34/L98H, TR46/Y121F/T289A, and M220 in the *Aspergillus fumigatus* (*A. fumigatus*) *Cyp51A* gene by subsequent DNA sequence analysis, we investigated in parallel the commercially available AsperGenius® real time PCR system in detecting the *Cyp51A* alterations TR34/L98H and Y121F/T289A directly from 52 clinical samples (15 biopsies, 22 bronchoalveolar lavage (BAL), 15 cerebrospinal fluid (CSF) samples) and ARAf isolates (*n* = 3) of immunocompromised patients. We analyzed DNA aliquots and compared both methods concerning amplification and detection of *Aspergillus* DNA and *Cyp51A* alterations. As positive control for the feasibility of our novel Y121F and T289A PCR assays, we used two *A. fumigatus* isolates with the TR46/Y121F/T289A mutation combination isolated from hematological patients with known C*yp51A* alterations and a lung biopsy sample of a patient with acute myeloid leukemia (AML). The rate of positive ARAf PCR results plus successful sequencing using the ARAf PCR assays was 61% in biopsies, 29% in CSF, 67% in BAL samples and 100% in isolates. In comparison the amount of positive PCRs using the AsperGenius® assays was 47% in biopsies, 42% in CSF, 59% in BAL samples and 100% in isolates. Altogether 17 *Cyp51A* alterations were detected using our ARAf PCRs plus DNA sequencing and therefrom 10 alterations also by the AsperGenius® system. The comparative evaluation of our data revealed that our conventional PCR assays are more sensitive in detecting ARAf in BAL and biopsy samples, whereby differences were not significant. The advantage of the AsperGenius® system is the time saving aspect. We consider non-culture based molecular detection of *Aspergillus* triazole resistance to be of high epidemiological and clinical relevance in patients with hematological malignancies.

## Introduction

*Aspergillus fumigatus* (*A. fumigatus*) is one of the major live-threatening fungal pathogens (Brown et al., 2012). It is estimated that more than 200,000 severe infections occur worldwide annually (Brown et al., 2012). Due to an increase in immunocompromised patients more people are at risk to suffer from invasive aspergillosis (IA) (Kim, 2016) which is associated with high mortality rates, especially in patients with malignant hematological diseases (Kontoyiannis et al., 2010; Perfect et al., 2014; Koehler et al., 2017). Triazoles are the main stay in the prophylaxis and treatment of IA.

The situation is worsened by an increasing prevalence of triazole resistant *Aspergillus* infections (Steinmann et al., 2015; van der Linden et al., 2015; Verweij et al., 2016; Garcia-Rubio et al., 2017) which is associated with a much higher mortality rate (van der Linden et al., 2011; Steinmann et al., 2015; Verweij et al., 2015; Chong et al., 2016; Meis et al., 2016). Triazole treatment failure was observed in 6/8 patients with a resistance associated mutation (RAM) compared with 12/45 patients without RAMs (*p* = 0.01). Six week mortality was 2.7 times higher in patients with RAMs (50 vs. 19%; *p* = 0.07) (Chong et al., 2016). About 50–80% of triazole resistance in *A. fumigatus* is caused by mutations in the *Cyp51A* gene (Dudakova et al., 2017). The 14-alpha-sterol-demethylase, the product of the *Cyp51A* gene, plays a major role in the ergosterol biosynthesis (Mellado et al., 2001), whereby triazoles act through inhibiting the 14-alpha-sterol-demethylase. The most frequent *Cyp51A* mutation combination found is the TR34/L98H gene alteration (Dudakova et al., 2017). In 2015 van der Linden et al. described the TR46/Y121F/T289A mutation combination as the second most frequent resistance-mechanism causing high level triazole resistance (van der Linden et al., 2015; van Ingen et al., 2015).

Due to mostly negative *Aspergillus* cultures from clinical material of hematological patients in microbiological diagnostics (De Pauw et al., 2008; Ruhnke et al., 2012; Morrissey et al., 2013; Koehler et al., 2017) and due to the higher mortality rates caused by azole resistant *Aspergillus fumigatus* (ARAf) infections, it is of high clinical impact to achieve sensitive and early detection of *A. fumigatus* including a triazole resistance. Therefore, molecular methods are required especially in hematological patients. Several polymerase chain reactions (PCR) assays for the detection of *A. fumigatus* and its *Cyp51A* mutations from clinical isolates have been published (Dudakova et al., 2017). Concerning the detection of *Aspergillus* DNA and triazole resistance mutations directly from primary clinical samples, the commercial real time PCR kit system AsperGenius® (Pathonostics; Maastricht, The Netherlands) is described to detect *Aspergillus* and four resistance-related mutations validated for bronchoalveolar lavage (BAL) and serum specimens (Chong et al., 2015, 2016; White et al., 2015b). The novel MycoGENIE® (Ademtech, Pessac, France) real time PCR kit is also able to identify *Aspergillus* DNA, but only the TR34/L98H mutation combination in serum and respiratory samples (Dannaoui et al., 2017).

Our group established TR34/L98H, TR46, and M220 ARAf PCR assays with subsequent DNA sequence analysis for the detection of the most common *Cyp51A* mutations that are correlated with triazole resistance in *A. fumigatus* directly from primary clinical samples. In addition, we recently developed two PCR assays for the detection of the Y121F and the T289A mutations from clinical samples that are associated with the TR46 tandem repeat. Blood, BAL, biopsy, and cerebrospinal fluid (CSF) samples were previously investigated and there four TR34/L98H and one TR46/Y121F/T289A *Cyp51A* mutations were successfully identified in five BAL and biopsy samples (Hamprecht et al., 2012; Rath et al., 2012; Spiess et al., 2012, 2014; Rössler et al., 2017).

In this study we compared the AsperGenius® kit system to our six in-house ARAf PCR assays with subsequent DNA sequence analysis concerning the sensitivity of detection of *Aspergillus* DNA and present triazole resistance mutations investigating 22 BAL and 15 CSF samples, 15 biopsies and three clinical ARAf isolates of immunocompromised patients.

## Materials and methods

### Patients

For the determination of mutations in the *A. fumigatus Cyp51A* gene conferring triazole resistance (TR34/L98H; TR46/Y121F/T289A and M220 alterations), we investigated clinical samples (BAL, tissue biopsies, CSF) of 52 immunocompromised patients mainly with hematological malignancies [AML *n* = 11; ALL *n* = 11; CLL *n* = 3; MDS *n* = 1; NHL *n* = 14; Hodgkin lymphoma *n* = 1; solid tumor *n* = 3; autoimmune neutropenia *n* = 2; immunosuppression not otherwise specified (NOS, *n* = 6)]. All samples were previously tested positive for *Aspergillus* DNA using our in-house diagnostic *Aspergillus* PCR assay (Skladny et al., 1999). Samples submitted to the scientific laboratory of the Department of Hematology and Oncology of the Mannheim University Hospital, Germany, for diagnosing IA were analyzed to elucidate PCR performance. Patients' data had been anonymized previously. Analyses were done according to Good Clinical Practice (GCP) guidelines as well as in concordance with the Declaration of Helsinki. The study was approved by the local Ethics Committee (Ethics Committee of the Faculty of Medicine Mannheim, University of Heidelberg, Germany; reference number 2011-280N-MA) and documented under ClinicalTrials.gov (identifier NCT01695512).

### Clinical samples

Bronchoscopy and BAL was performed according to standardized operating procedures as described elsewhere (Skladny et al., 1999), and BAL samples were obtained in a sterile vessel without conservation media. The mean sample volume was 10 mL. Tissue samples were obtained by needle biopsies (lung, liver, kidney) or surgical procedures (brain, other samples) under sterile conditions. Cerebrospinal fluid was gained and prepared as described (Hummel et al., 2006). We examined 55 specimens in total; these included 22 BAL specimens, 15 biopsies, 15 CSF specimens, and three ARAf isolates which served as positive controls.

### Strains and growth conditions

*A. fumigatus* wildtype strain (DSM 819) was obtained from the Deutsche Sammlung von Mikroorganismen und Zellkulturen GmbH, Braunschweig, Germany, and the Institute of Medical Microbiology and Hygiene, Mannheim University Hospital, Mannheim, Germany.

One triazole-resistant TR34/L98H positive *A. fumigatus* clinical isolate was obtained from the University Hospital of Cologne, Cologne, Germany. The isolate showed the following MIC (minimum inhibitory concentration) values obtained by EUCAST reference microdilution method: voriconazole 2.0 mg/L, itraconazole >16.0 mg/L, posaconazole 0.5 mg/L (Hamprecht et al., 2012). The second triazole resistant TR46/Y121F/M172I/T289A positive *A. fumigatus* strain was from the Institute for Medical Microbiology, University Medical Center Göttingen, Göttingen, Germany (originated from the Institute of Medical Microbiology and Hygiene, Technische Universität Dresden, Dresden, Germany) and showed the following EUCAST MIC values: voriconazole >32 mg/L, itraconazole 1 mg/L, posaconazole 0.5 mg/L (Rössler et al., 2017). Characterization of the third multi-azole resistant TR46/Y121F/T289A positive clinical isolate (IMMi2107) from the Institute of Medical Microbiology, University Hospital Essen, Essen, Germany revealed the following EUCAST MIC values: itraconazole >16.0 mg/L, voriconazole 2 mg/L, posaconazole 0.5 mg/L (Steinmann et al., 2015).

### DNA extraction

DNA extraction from fungal cultures and from biopsy, CSF, and BAL samples was performed using the phenol/chloroform extraction method as previously described (Sambrook et al., 1989; Skladny et al., 1999). Tissue samples were processed additionally in liquid nitrogen for disruption. The tissue was sheared using a scalpel in a sterile petri dish under sterile conditions. The generated nuggets were transferred into a tissueTUBE™ used for processing of the sample in a cryoPREP™ workflow (Covaris; USA). The tissueTUBE™ containing the tissue material was incubated in liquid nitrogen for 30–45 s until the sample was completely frozen. After freezing the tube was fitted into the cryoPREP™ workflow, were the tissue was pestled. The frozen tissue pieces were transferred into a sterile 50 ml reaction tube and mixed with 1.5 ml 1x PBS buffer. The tissue/PBS mixture was transferred into a 1.5 ml reaction tube and centrifuged at 13,000 rmp for 10 min. Supernatant was discarded and the pellet was resuspended in 250 μl 1x PBS buffer.

### ARAf PCR assays

**Figure 1:**
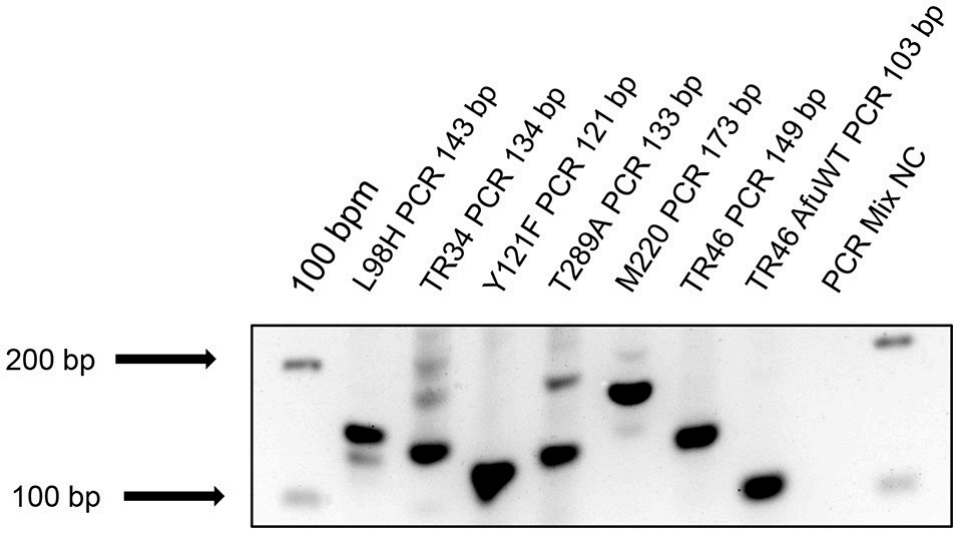
Agarose gel electrophoresis of the six established in-houes ARAf PCR assays. L98H, TR34, Y121F, T289A, and M220 PCRs were performed using DNA (mixture of human and fungal DNA) of a TR34/L98H mutation positive BAL sample of a patient suffering from AML (Hamprecht et al., 2012). The TR46 PCR was performed using a TR46 positive clinical *A. fumigatus* isolate (lane 7) and *A. fumigatus* wildtype (WT) DNA (lane 8). NC, negative control, bpm, base pair marker.

All 52 clinical specimens plus the 3 isolates were analyzed using our six in-house ARAf PCR assays. Our modified one-step L98H-PCR assay amplifies a 143 bp fragment and has been previously described in Spiess et al. (2014). The TR34 nested PCR assay, as well as the M220 one-step PCR-assay have been described in 2012, amplifying a 100 bp and a 173 bp DNA-fragment, respectively (Spiess et al., 2012). To complete our TR46 PCR assay (Spiess et al., 2014), we established new PCR assays to detect the corresponding Y121F and T289A mutations. Additionally, we slightly adjusted our established TR46 PCR assay (Spiess et al., 2014) by lowering the annealing temperature to 50°C instead of 52°C. The in-house ARAf PCR assays are summarized in Table 1 and shown in Figure 1.

**Table 1:** Summary of the six established in-house ARAf PCR assays.

**Mutation**	**Fragment length**	**PCR assay**	**Sensitivity**
L98H (Spiess et al., 2012)	143 bp	One-Step	6 pg
TR34 (Spiess et al., 2012)	1st step: 235 bp (WT) 2nd step:100 bp (WT)	Two-Step	600 fg
M220 (Spiess et al., 2012)	173 bp	One-Step	4 pg
L98H (Spiess et al., 2014)	143 bp	One-Step	300 fg
TR46 (Spiess et al., 2014)	1st step: 213 bp (WT) 2nd step:103 bp (WT)	Two-Step	300 fg
Y121F	121 bp	One-Step	300 fg
T289A	133 bp	One-Step	300 fg

*WT, wild type*.

The *A. fumigatus Cyp51A* gene specific Y121F and T289A primer pairs were designed from the *A. fumigatus Cyp51A* DNA sequence (AF 338659.1) available in the GenBank database (http://www.ncbi.nlm.nih.gov/). To predict the potential cross reactivities of the *A. fumigatus Cyp51A* primer sequences with human genomic DNA sequences, additional database searches were performed by using the primer-BLAST service. The melting temperatures (T_m_s) of the primers and possible secondary structures were calculated using also the NCBI primer designing tool primer-BLAST 2016 (http://www.ncbi.nlm.nih.gov/). The synthetic oligonucleotides were commercially synthesized (Sigma, Munich, Germany) and diluted to 100 μM in ddH_2_O.

### Novel ARAf PCR assays, specificity and sensitivity

The novel PCR assays were performed as one-step PCR assays. The generated PCR fragment for the Y121F assay was 121 bp in length using the primer pair *Cyp51A*-Y121F-s1 (5′-CATTGACGACCCCCGTTTTC-3′) and *Cyp51A*-Y121F-as1 (5′-GGCACATGAGACTCTAACGCA-3′). The generated PCR fragment for the T289A assay was 133 bp in length using the primer pair *Cyp51A*-T289A-s1 (5′-CACATACAAAAACGGCCAGCA-3′) and *Cyp51A*-T289A-as1 (5′-TTTTGGCTGTGAGGCCAGTC-3′). The following PCR conditions were used for both PCR reactions: total volume, 25 μl; 3 μl template DNA (~100 ng human DNA plus an unknown amount of *A. fumigatus* DNA), 3 mM MgCl_2_, 0.25 mM each deoxynucleoside triphosphate, 1 U of *Taq* polymerase (Invitrogen GmbH, Karlsruhe, Germany), 20 pmol of each primer; DNA thermal cycler, 5 min of initial denaturation at 94°C, 40 cycles of 94°C for 45 s, 54°C for 1 min, 72°C for 1 min, after the 40 cycles 94°C for 45 s, 54°C for 45 s, and final extension at 72°C for 10 min. Sensitivity of the novel PCR assays was determined using serially diluted *A. fumigatus* wildtype DNA as template. To test cross reactivity of the designed primer pairs with human genomic DNA, we investigated a sample adopted in the PCR assays containing a mixture of 100 ng human genomic DNA and 50 ng of *A. fumigatus* wildtype DNA. PCR products were analyzed by agarose gel analysis stained with GelStar (Bio-Rad GmbH, Munich, Germany). The detection threshold for both assays was 300 fg of genomic *A. fumigatus* DNA.

### Control

The established PCR assays for the detection of Y121F/T289A directly from clinical samples (BAL, tissue biopsies, CSF) as a marker of the TR46/Y121F/T289A genotype were tested using DNA of a TR46/Y121F/T289A positive, multi-azole resistant clinical isolate (IMMi 2107).

### Sequence analysis

The PCR products were used directly for mandatory sequence analysis. The PCR products were purified using the MiniElute PCR purification kit (Qiagen, Hilden, Germany) and a minimum of 50 ng DNA was sequenced (Sequiserve, Vaterstetten, Germany). To detect potential mutations in the PCR products analyzed by DNA sequence analysis, the sequence of the products was compared to the sequence of the *A. fumigatus Cyp51A* wildtype sequence using the NCBI alignment service AlignSequenceNucleotideBlast (http://www.ncbi.nlm.nih.gov/) and the FunResDB-A (Weber et al., 2018).

### AsperGenius® PCR kit system

The AsperGenius® system is a real time multiplex PCR approach. The system was used for the identification of prevalent mutations conferring resistance against triazoles in clinical samples tested positive for *Aspergillus* DNA before using our in-house diagnostic *Aspergillus* PCR assay (Skladny et al., 1999).

**Figure 2:**
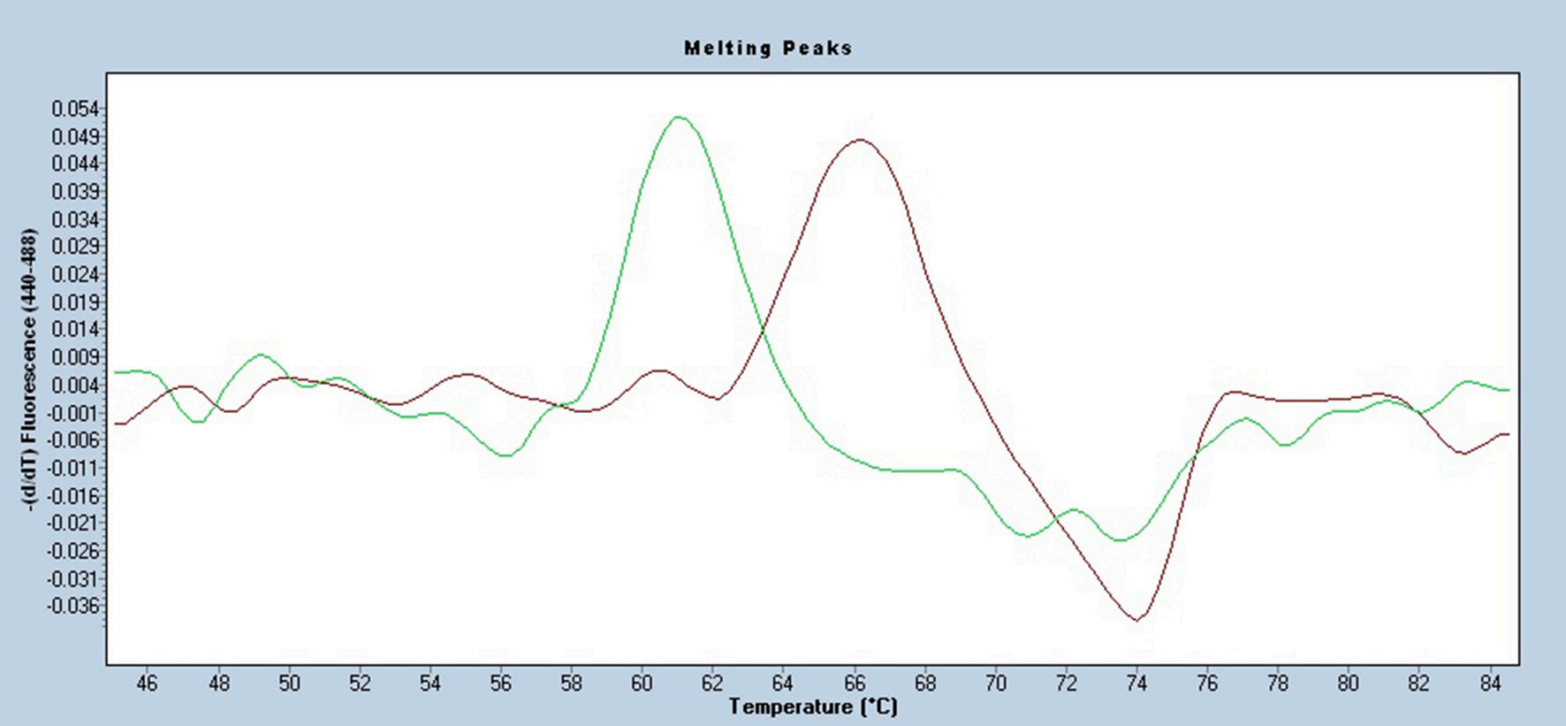
AsperGenius® based melting curve analysis of a potentially present L98H mutation in a BAL sample containing *A. fumigatus* DNA. The sample contained *A. fumigatus* wild type DNA (green curve in the range 61.0–64.0°C) compared to the L98H positive control DNA (brown curve in the range 65.5–68.5°C).

The AsperGenius® system contains both a diagnostic *Aspergillus* DNA detection kit and a kit for detection of four triazole resistance mutations, namely TR34, L98H, Y121F, and T289A. The species multiplex assay allows the specific detection of *A. fumigatus* complex, *Aspergillus terreus*, and other *Aspergillus* species by targeting the 28S rRNA multicopy gene. The AsperGenius® resistance multiplex assay targets the single copy *Cyp51A* gene of *A. fumigatus* and detects the TR34, L98H, Y121F, and T289A mutations to differentiate wild type from mutant *A. fumigatus*. The different *Cyp51*A alterations are detected by melting curve analysis in different fluorescence detection channels (450, 530, 598, and 645 nm) by a shift in the melting curves of the mutation-bearing DNA compared to wild type DNA. We tested the approach using the LightCycler 480 technology (Roche Diagnostics GmbH, Mannheim, Germany) and only analyzed the triazole resistance detection in clinical BAL, biopsy, and CSF samples. One representative analysis of a BAL sample for the detection of the L98H mutation by melting curve analysis is shown in Figure 2.

### Statistics

Statistical calculations were performed using the Chi-square test.

## Results

Four established (Spiess et al., 2012, 2014) and two novel in-house ARAf PCR assays were compared to the commercially available AsperGenius® kit system concerning the detection of *A. fumigatus* DNA and *Cyp51A* key mutations directly from clinical samples of immunocompromised patients. Fifty-two clinical samples (15 biopsies, 22 BAL, 15 CSF samples) of 52 immunocompromised patients and three *A. fumigatus* isolates were investigated with both methods and results were compared.

Analyzing 22 BAL specimens with our six Mannheim ARAf PCR assays revealed the following results: 82% (18/22) of the samples showed a positive signal in the TR34 PCR assay, 73% (16/22) were sequenced successfully with one TR34 mutation detected. The AsperGenius® PCR kit system showed positive signals in 64% (14/22) of the cases and discovered also the TR34 mutation. In the ARAf L98H PCR assay we received positive signals in 91% (20/22) of the cases, in 77% (17/22) the sequence analysis was effective and one L98H mutation was detected. This mutation was also detected by the AsperGenius® kit system, but only 31% (7/22) positive L98H PCR results were achieved using this system. Considering the in-house Y121F and T289A assays, positive signals were obtained in 68% (15/22) and 59% (13/22) of the cases, respectively. From 13/22 (59%) and 11/22 (50%) PCR reactions sequence analysis was also possible. No Y121F/T289A mutations were revealed. In 72% (16/22) of the samples, the Y121F PCR showed positive signals in the AsperGenius® system, the T289A PCR assay was positive in 68% (15/22) of the cases. In both approaches no mutations were detected. The in-house TR46 PCR assay, which is not represented in the AsperGenius® PCR kit, showed positive signals in 77% (17/22) of the cases with 73% (16/22) successful DNA-sequencing. No TR46 alterations were detected in BAL specimens. The in-house M220 PCR assay was successful in 68% (15/22) of the cases; all of them were sequenced without the detection of another mutation. This mutation is also not represented in the AsperGenius® PCR kit. Overall 74% (98/132) of our in-house ARAf PCRs showed positive signals accompanied by 67% (88/132) of successful sequencing, whereas 59% (52/88) of the AsperGenius® PCR assays were positive for the detection of *A. fumigatus* DNA from BAL samples. Statistical analysis revealed that the difference between 59 and 67% sensitivity was not significant (*p* = 0.252). One BAL sample revealed the TR34/L98H mutation combination in both systems. This BAL sample was obtained from a neutropenic AML patient characterized first in 2012 (Hamprecht et al., 2012).

Of the biopsies, 69% (62/90) showed positive signals in our ARAf PCR assays, of these 61% (55/90) were sequenced successfully. In contrast, 47% (28/60) of the AsperGenius® PCRs showed positive results, revealing one Y121F and one T289A mutation. Via sequence analysis we were additionally able to detect those two mutations as well as the corresponding TR46 alteration. The difference in sensitivity from biopsies was also not significant (*p* = 0.081). The TR46/Y121F/T289A mutation combination was found in a lung biopsy sample of a patient suffering from AML and described in Rössler et al. (2017). Furthermore, we detected three L98H mutations and one TR34 alteration using our in-house ARAf PCRs with subsequent DNA sequencing. One L98H/TR34 mutation combination was found in a brain biopsy of a patient suffering from ALL and another L98H mutation in a lung biopsy of an AML patient (Spiess et al., 2012, 2014). An additional L98H mutation not yet described was found in a lung biopsy of an osteosarcoma patient. The potentially present corresponding TR34 alteration in this patient was not detectable due to technical reasons. The TR34 ARAf PCR approach provided no positive PCR signal applicable for DNA sequence analysis. None of these mutations were detected by the AsperGenius® system from biopsies.

In CSF specimens, the AsperGenius® system showed better results with 42% (25/60) positive PCRs. The in-house ARAf PCR assays were positive in 39% (34/87) of the cases with only 29% (25/87) successful sequence analysis. The difference in sensitivity between both assays was not significant (*p* = 0.104). No mutations were detected from CSF samples with both methods. Isolates showed 100% positive fungal DNA detection in the in-house ARAf PCRs as well as in the AsperGenius® PCR kit. The known present TR46/Y121F/T289A mutation combinations were found with both methods. With our approach we were able to detect nine mutations directly from clinical samples. Due to its setup the AsperGenius® kit was not able to detect any TR46 alteration. Furthermore, the system did not detect three L98H mutations and one TR34 alteration from biopsy samples.

**Figure 3:**
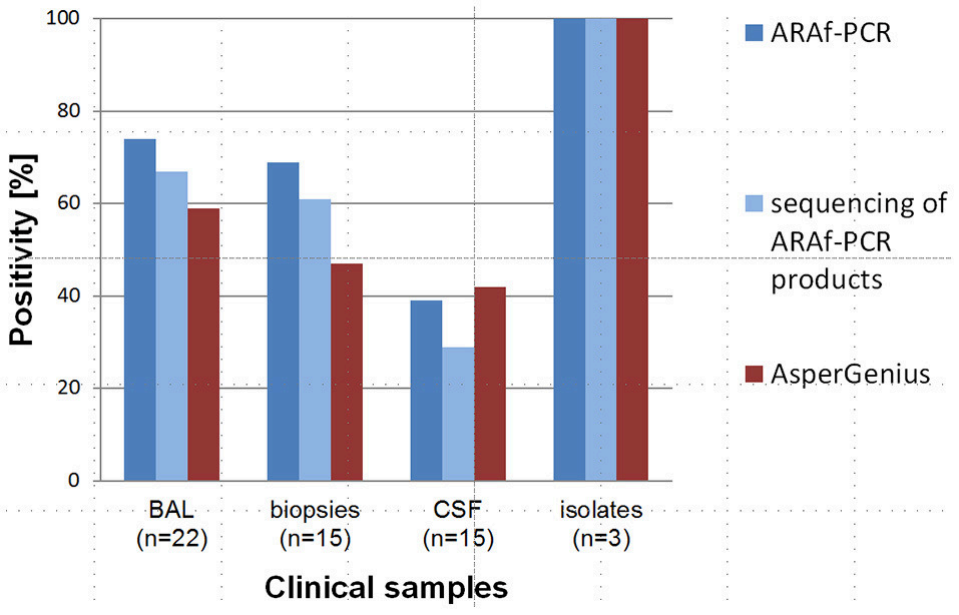
Comparison of diagnostic results of our ARAf PCR assays and the AsperGenius® system concerning the detection of *A. fumigatus* DNA directly from clinical samples as a prerequisite for the characterization of triazole resistance mutations. ARAf PCR results are shown in the dark blue bars, positive ARAf PCR sequencing is shown in light blue bars; the red bars show the AsperGenius® PCR results. In BAL samples the positivity of the ARAf PCRs plus sequencing was slightly higher (8%). In biopsy samples the ARAf PCRs assays plus sequencing showed a 14% higher positivity. Results in CSF (cerebrospinal fluid) samples were nearly identical, whereby after including sequencing results, the sensitivity of the in-house ARAf system decreased to 29%. The amplification of DNA from isolates was 100% for both methods. The detected differences concerning sensitivity of the assays were not statistically significant (BAL, *p* = 0.252; biopsies, *p* = 0.081; CSF, *p* = 0.104).

Detailed information about positivity of the assays in the different clinical specimens and detected *Cyp51A* mutations can be seen in Tables 2, 3. A comparison of the results for both methods is shown in Figure 3.

**Table 2:** Summary of the comparison of positive diagnostic results of ARAf PCR assays and the AsperGenius® system concerning the detection of *A. fumigatus* DNA and *Cyp51A* mutations directly from clinical samples.

		**In-house ARAf PCR**			**AsperGenius^®^**	
		**PCR+**	**Successful sequencing**	**Mutation+**	**PCR+**	**Mutation+**
BAL	TR34	82% (18/22)	73% (16/22)	1	64% (14/22)	1
	L98H	91% (20/22)	77% (17/22)	1	31% (7/22)	1
	TR46	77% (17/22)	73% (16/22)	0	–	–
	Y121F	68% (15/22)	59% (13/22)	0	72% (16/22)	0
	T289A	59% (13/22)	50% (11/22)	0	68% (15/22)	0
	M220	68% (15/22)	68% (15/22)	0	–	–
	Total	74% (98/132)	67% (88/132)	2	59% (52/88)	2
BIOPSY	TR34	53% (08/15)	53% (08/15)	1	33% (05/15)	0
	L98H	67% (10/15)	60% (09/15)	3	33% (05/15)	0
	TR46	60% (09/15)	53% (08/15)	1	–	–
	Y121F	80% (12/15)	67% (10/15)	1	60% (09/15)	1
	T289A	80% (12/15)	60% (09/15)	1	60% (09/15)	1
	M220	73% (11/15)	73% (11/15)	0	–	–
	Total	69% (62/90)	61% (55/90)	7	47% (28/60)	2
CSF	TR34	33% (5/15)	27% (4/15)	0	47% (7/15)	0
	L98H	40% (6/15)	40% (6/15)	0	40% (6/15)	0
	TR46	21% (3/14)	21% (3/14)	0	–	–
	Y121F	53% (8/15)	40% (6/15)	0	40% (6/15)	0
	T289A	50% (7/14)	14% (2/14)	0	40% (6/15)	0
	M220	36% (5/14)	29% (4/14)	0	–	–
	Total	39% (34/87)	29% (25/87)	0	42% (25/60)	0

**Table 3:** Summary of all detected *Cyp51A* mutations from clinical samples and isolates so far.

**Clinical samples and isolates**	**In-house ARAf PCRs plus sequencing**						**AsperGenius^®^**			
	**TR34**	**L98H**	**TR46**	**Y121F**	**T289A**	**M220**	**TR34**	**L98H**	**Y121F**	**T289A**
BAL: (AML) (Hamprecht et al., 2012; Spiess et al., 2014)	+	+	–	–	–	–	+	+	–	–
Lung biopsy: (AML) (Spiess et al., 2014)	–	+	–	–	–	–	–^*^	–^*^	–^*^	–^*^
Brain biopsy: (ALL) (Spiess et al., 2012)	+	+	–	–	–	–	–^*^	–^*^	–	–
Lung biopsy: (Osteosarcoma)	–^*^	+	–^*^	–	–^*^	–	–^*^	–^*^	–^*^	–^*^
Lung biopsy: (AML) (Rössler et al., 2017)	–	–	+	+	+	–	–^*^	–^*^	+	+
Isolate of lung biopsy: (AML) (Rössler et al., 2017)	–	–	+	+	+	–	–	–	+	+
Isolate of BAL: (AML) (Hamprecht et al., 2012)	+	+	–	–	–	–	+	+	–	–
Isolate (IMMi 2107): (Steinmann et al., 2015)	–	–	+	+	+	–	–	–	+	+

*Both methods found all known present mutations in isolates. The ARAf PCRs plus DNA sequencing detected nine mutations and the AsperGenius® assay four mutations directly from primary clinical samples. AML, acute myeloid leukemia; ALL, acute lymphoid leukemia; PC, positive control*.

* *DNA not amplified*.

## Discussion

Diagnosis of IA in hematological high risk patients often remains largely unsatisfying, especially since *Aspergillus* culture remains mostly negative in microbiological diagnostics in this group of patients (Ruhnke et al., 2012). In the SEPIA study—a prospective multicenter cohort study in hematological and oncological centers in Germany−179 of 3,067 patients with acute leukemia were diagnosed suffering from IA, among these 96% were classified as probable IA following the EORTC/MSG consensus criteria (De Pauw et al., 2008; Koehler et al., 2017). Only in 14% of these cases *A. fumigatus* was proved in culture (Koehler et al., 2017), reflecting the fact that culture-based IA diagnostics alone is not sufficient in these patients. Studies already indicate that molecular-based detection methods in addition to culture-based diagnostics are beneficial for the outcome of the patients (Rickerts et al., 2007; Guegan et al., 2017); this applies especially to hematological patients showing poorer test-performances with culture-based diagnostic approaches (Guegan et al., 2017).

In this study we compared the commercially available AsperGenius® kit system to our six in-house ARAf PCR assays with subsequent DNA sequence analysis concerning the sensitivity of detection of *Aspergillus* DNA and triazole resistance mutations investigating 22 BAL and 15 CSF samples, 15 biopsies, and three clinical isolates of immunocompromised patients.

We included BAL, CSF and biopsy specimens from the site of infection and explicitly excluded blood samples. At the time of diagnostics for IA most patients at high risk for IPA in Germany are already undergoing antifungal prophylaxis or early pre-emptive antifungal therapy. Springer et al. demonstrated in 2016 that *Aspergillus* PCR had a high false predictive value in patients during antifungal medication (Springer et al., 2016). Furthermore, the diagnostic *Aspergillus* PCR showed a better test performance in BAL than in peripheral blood samples (Boch et al., 2016). Nevertheless, White et al. have examined the diagnostic value of the diagnostic AsperGenius® kit system in serum and plasma samples (White et al., 2015b, 2017). For serum samples a sensitivity of 79% with a specificity of 100% was reached, whereas in plasma samples sensitivity and specificity of 80 and 78% was observed (White et al., 2015b, 2017). In other PCR assays plasma specimens were superior compared to serum specimens (White et al., 2015a). In all studies, patients have been for the most part under antifungal prophylaxis.

For the comparison of the triazole resistance AsperGenius® kit system to our in-house ARAf PCR assays we used the DNA extraction methods described in our previous publications (Skladny et al., 1999; Hummel et al., 2006; Spiess et al., 2012). The extraction method differs from the one depicted in the manual of the AsperGenius® kit system (BioMerieux EasyMag extraction method). This way was chosen due to the fact that DNA of several clinical samples had already been extracted by the time the study started and there was no raw material left over. We have not yet evaluated the impact of the extraction methods on the performance of the AsperGenius® kit system.

The comparative evaluation of the generated data revealed that our in-house ARAf PCR assays are more sensitive for the analysis of BAL and biopsy samples, although the calculated differences were not statistically significant. Nevertheless, carrying out six PCR assays with subsequent DNA sequence analysis is time consuming. Regarding this point the AsperGenius® kit system has an advantage over our in-house ARAf PCR assays. In case of CSF samples both approaches showed no convincing results, with AsperGenius® being lightly more sensitive. Most likely negative results in biopsy samples in the AsperGenius® kit system could be caused by interference with human DNA during the PCR reactions.

Unsuccessful DNA sequencing of the PCR fragments generated by the in-house ARAf PCRs could be due to the low amount of *A. fumigatus* DNA in the clinical samples and therefore to the low amount of DNA generated by the PCR assays. By agarose gel electrophoresis 25 pg of DNA are visible, for the performance of Sanger sequencing, 50 ng of fungal DNA are necessary. Both systems showed a 100% sensitivity when investigating *A. fumigatus* isolates, because in this scenario the amount of fungal DNA and the interference with human DNA are no limiting factors. The reason for the statistically insignificant calculated differences in the determination of the sensitivity of the two test systems may be due to the number of samples investigated; the number of investigated *Aspergillus* DNA positive clinical samples is owing both to the low prevalence of IA in hematological high risk patients during antifungal therapy and the low prevalence of *Aspergillus* triazole resistance in Germany.

Meanwhile MycoGENIE®, a new commercial kit has been released. Like AsperGenius® it offers the detection of *Aspergillus* DNA as well as the verification of *Cyp51A* mutations. In contrast to AsperGenius® which detects up to four mutations in the *Cyp51A* gene MycoGENIE® is only able to detect TR34 and L98H mutations (Dannaoui et al., 2017).

Non-culture based molecular detection methods of *A. fumigatus* triazole resistance directly from the site of infection are extremely important in hematological patients at high risk for IA. This can be underlined by the fact that molecular detection methods have the ability to detect triazole-susceptible and triazole-resistant coinfections. Cultural diagnosis may miss these coinfections resulting in insufficient therapy (Schauwvlieghe et al., 2017). To open the possibility to include *Aspergillus* PCR in the revised European Organization for Research and Treatment of Cancer/Invasive Fungal Infections Cooperative Group and National Institute of Allergy and Infectious Diseases Mycoses Study Group (EORTC/MSG) definitions for fungal disease, further optimized commercially manufactured assays may be required to provide standardization and accessibility.

Therefore, a prospective study comparing conventional PCR assays from clinical material of the site of infection to commercial kits like AsperGenius® and if possible cultural results in a larger cohort of hematological and oncological patients is ongoing.

## Author contributions

PP contributed to the search of scientific literature, data generation and collection, data analysis, and writing of the manuscript. JuS performed the experiments, contributed to data analysis, and writing of the manuscript. TB contributed to reading and editing of the manuscript, and to data analysis. OC, AH, P-MR, JöS, OB, and TM contributed to material collection and editing and writing of the manuscript. AD and NM performed the experiments. W-KH contributed to supervision and editing of the manuscript. DB and BS contributed to search of scientific literature, trial design, data collection, data interpretation, and writing of the manuscript.

### Conflict of interest statement

OC has received research grants from Actelion, Amplyx, Arsanis, Astellas, AstraZeneca, Basilea, Bayer, Cidara, Duke University (NIH UM1AI104681), F2G, Gilead, GSK, Leeds University, Matinas, Medicines Company, MedPace, Melinta, Merck/MSD, Miltenyi, Pfizer, Rempex, Roche, Sanofi Pasteur, Scynexis, Seres, is a consultant to Amplyx, Actelion, Astellas, Basilea, Cidara, Da Volterra, F2G, Gilead, Janssen, Matinas, Menarini, Merck/MSD, Paratek, PSI, Scynexis, Seres, Summit, Tetraphase, Vical, and received lecture honoraria from Astellas, Basilea, Gilead, Merck/MSD, and Pfizer. AH has received a travel grant from Astellas and educational lecture honoraria from MSD, Pfizer, and Astellas. JSt received honoraria for lectures and advisory boards from Gilead Sciences and TEVA. DB is consultant to Basilea, Gilead Sciences, Merck Sharp & Dohme/Merck, received research grants from Gilead Sciences and Pfizer, serves on the speakers' bureau of Astellas, Basilea, Gilead Sciences, Merck Sharp & Dohme/Merck, Pfizer, and TEVA, and received travel grants from Astellas, Gilead Sciences, Merck Sharp & Dohme/Merck, and Pfizer. The other authors declare that the research was conducted in the absence of any commercial or financial relationships that could be construed as a potential conflict of interest.
